# Comparison of Cytotoxicity of Four Different Adhesive Materials Before and After Polymerisation

**DOI:** 10.3290/j.ohpd.a43940

**Published:** 2020-04-01

**Authors:** Suzan Cangul, Ozkan Adiguzel, Samet Tekin

**Affiliations:** a Assistant Professor, Department of Restorative Dentistry, Faculty of Dentistry, Dicle University, Diyarbakir, Turkey. Hypothesis, experimental design, wrote the manuscript.; b Professor, Department of Endodontics, Faculty of Dentistry, Dicle University, Diyarbakir, Turkey. Contributed substantially to Materials and Methods and Discussion sections.; c Assistant Professor, Department of Prosthodontics, Faculty of Dentistry, Firat University, Elazığ, Turkey. Contributed substantially to Discussion section.

**Keywords:** adhesive materials, cytotoxicity, polymerisation, MTT test

## Abstract

**Introduction:**

The aim of this study was to make a quantitative comparison of the cytotoxic potentials of four different polymerised and unpolymerised self-etching adhesives which were newly used clinically.

**Materials and Methods:**

Cytotoxic effects of both polymerised and unpolymerised forms of all test adhesives were evaluated against L929 cell line using the MTT test. The activity for unpolymerised adhesives was assessed in different doses and incubation times manner. On the other hand, cytotoxicity of the polymerised adhesives prepared at different extraction times were evaluated as dependent on incubation times. Two-Way Variance Analysis and Bonferroni post-test was used for statistical evaluation.

**Results:**

There were statistically significant differences between the groups (p < 0.05). In general, it was shown that unpolymerised and polymerised forms of each of the test compounds exhibited a time-dependent cytotoxic effect. However, the effect on polymerised forms was found to be independent of the duration of the extraction, while the effect on the unpolymerised forms increased dose-dependently. It was also determined that the most cytotoxic material in the unpolymerised form was Dentsply and in the polymerised form was Tokuyama.

**Conclusions:**

Dentsply should be preferred over Tokuyama to be able to provide clinically long-lasting restorations.

Together with the developments in modern-day dentistry, several aesthetic and long-lasting new materials have been developed for the protection of healthy dental tissue.^5^ These are intended to meet patients’ aesthetic expectations and prevent problems such as microleakage, polymerisation freezing and the formation of secondary decay.^26^

Successful restoration of lost dental tissue has been achieved using invasive techniques with newly developed adhesive systems. The basic aim in the use of these materials is to form a strong bond between the resin and the tooth structure, to prevent leakage forming in the tooth–resin interface and to strengthen the retention of the restorative materials.^18^ However, several stages are necessary in the clinical application of these systems because of sensitivity. Several new systems with simple application stages have been recently developed to prevent failures.^5,26^

One-step self-etch adhesives, which have been recently produced for this purpose and are frequently used, are systems in which acidic monomer, primer and bonding agent are combined in a single flask.^20,24^ These adhesives contain ionic and hydrophilic monomers at high concentrations and are extremely hydrophilic. However, the combination of hydrophilic and hydrophobic monomers in a single flask provides a variable chemical state of the adhesives due to the low pH and various additive substances.^13^

The content of adhesive systems is formed of low-viscosity hydrophobic monomers such as Bis-GMA (bisphenol glycidyl dimethacrylate), UDMA (urethane dimethacrylate) and TEG-DMA (triethylene glycol dimethacrylate). Hydrophilic monomers such as hydroxyethylmethacrylate (HEMA) are added to increase infiltration to these agents.^6,25^

In the last 20 years, several innovations have been developed to increase the efficacy of these adhesive systems. When the currently used adhesive systems are compared with those of the past, the use and physical characteristics can be seen to have improved.^2^ In addition to these properties, it is also important that these systems, which are in close contact with dentine, are biocompatible in order to show clinically successful results.

Biocompatibility is defined as a biomaterial that is in contact with live tissues not creating toxicity, allergy, mutagenic or carcinogenic effects on other tissues of the body.^21^ As biocompatibility is a dynamic and continuing situation, it is dependent not only on the area where applied but also on the type of material and the function expected from it. A change in the conditions where the tissue and material are located disrupts the dynamics between them and a material that was initially biocompatible may become bio-incompatible over time.^14^

While developing new adhesive systems, the toxic effects of different substances in their structure are ignored. Knowledge of the cellular structural properties of the components of adhesive systems is extremely important in respect of biocompatibility. The cytotoxicity of the components in the contents of adhesive systems on tooth tissues should be investigated. Various methods are used for this purpose. These stages are as important as the development of new materials. Cell culture studies have shown that when these components come into direct contact with fibroblasts they are highly toxic. Moreover, the toxic levels of these components may change depending on the amount populating the dentin and accumulating in the pulp.^28^

In the other study, the researchers investigating the cytotoxicity of monomers on rat fibroblasts, the element with the highest toxic value was found to be 2,2-bis [4¢-(x-hydroxy-3¢-methacryloyoxy)phenyl] propane (bis-GMA), followed by urethane dimethacrylate (UDMA), triethylene-glycoldimethacrylate (TEGDMA) and 2-hydroxyethyl methacrylate (HEMA). In another study by Geurtsen et al^8^ of human gingival fibroblasts and periodontal ligament cells, TEGDMA was determined to be less cytotoxic than UDMA with the lowest toxicity values in human dental pulp cells.

The number of cells, membrane permeability, cell morphology and changes in intracellular metabolism of restorative materials are examined in cytotoxicity studies. In previous studies, cytotoxicity has been determined in several bonding agents.

Histopathologically, a bonding agent may induce pulp inflammation.^15^ The reason for the onset of inflammation in the pulp is the diffusion from the dental tubules of monomers that have remained unpolymerised following light polymerisation. These unpolymerised monomers can interact with odontoblasts and pulp cells. Monomers released from dental resin materials may cause adverse biological effects in mammalian cells (6-Fuang HM). Inflammatory reactions can be started in the pulp by these monomers, which can also be defined as antigens. The first cells affected by the elements expressed are the odontoblast cells below the circumferential dentine. Therefore, they are the most appropriate targets for cytotoxicity tests of different adhesive systems.^13^

In recent years, several new techniques have been developed to be able to conduct in vitro studies of cytotoxicity.^11^ The vast majority of these are cell culture tests that have usually used fibroblast cells. The use of these types of tests is suitable for the evaluation of biocompatibility, they can be repeated and have provided verifiable results.^23^

To be able to make an accurate evaluation in respect of cytotoxicity, the study model must be able to fully reflect the clinical environment. Two basic strategies are used in cytotoxic examination. The first is that the components of the material to be examined must be evaluated in a single layer in the culture environment. Then, by creating dose-response curves, the cytotoxicity can be examined. The second strategy is to mimic the structures that present barriers between the material and the cells.^29^

When previous studies are examined it can be seen that there is insufficient information about the cytotoxicity of one-step self-etch adhesive systems and there are few studies that have compared the cytotoxicity values of these systems before and after polymerisation. The aim of this study was to evaluate the cytotoxicity of four different one-step self-etch adhesive systems on fibroblast cells before and after polymerisation.

## Materials and Methods

### Test Materials

All the stages of this study evaluating the in vitro cytotoxic potential of four different adhesive materials shown in Table 1 were conducted within the TS EN ISO 10993 standards (TS EN ISO 10993-1, TS EN ISO 10993-12 and TS EN 10993-5).

**Table 1:** Materials tested by in vitro cytotoxicity method

Adhesive materials	Composition
3M-Espe Single Bond (3M-E)	MDP Phosphate Monomer, Dimethacrylate resins, HEMA, Vitrebond Copolymer, Filler, Ethanol, Water, Initiators, Silane
G-Premio Bond (GPB)	Three functional monomers (4-MET, MDP, MDTP)
Tokuyama Universal Bond (TUB)	Phosphoric acid monomer (New 3D-SR monomer), MTU-6, HEMA, Bis-GEMA, TEGDMA, Aseton BOND B: Borat, Peroksit, Aseton, İzopropil alkol, Su, MPTES
Dentsply-Sirona Prime&Bond Universal (PBU)	Phosphoric acid modified acrylate resin, Multifunctional acrylate, Bifunctional acrylate, Acidic acrylate, Isopropanol, Water, Initiator, Stabiliser

Unpolymerised adhesive systems tested in the study were sterilised by passing them through 0.2 μm filters after preparing 0.1% stock solutions in Dulbecco’s Modified Eagles medium (DMEM; Hyclone, Utah, USA) for maximum dissolution. Test concentrations (0.1%, 0.01% and 0.001%) for unpolymerised form of materials were prepared by serially diluting the samples collected from this stock concentration three times in 1/10 ratio in the culture medium.

For each adhesive material, 6 mm diameter 1 mm high specimens were prepared in sterile Teflon moulds in the laminar flow in accordance with the manufacturer’s recommendations. Tokuyama Universal Bond (TUB) was polymerised with air during application and the other three bonds were polymerised by light emitting diode (LED; Woodpecker, Guilin, Guangxi, China). 3M-E and G-Premio Bond (GPB) were treated with LED at 10 s, Prime&Bond Universal (PBU) at 20sn. Following the polymerisation step, adhesive materials were extracted in DMEM for 6 h, 1 days, 3 days and 7 days. Each extract was sterilised by passing it through 0.2 μm filters after extraction times.

### Cell Culture Study

In the MTT test to quantitatively evaluate the cytotoxic potential of the materials, the cell line used was formed from L929 rat fibroblasts derived from fibroblast roots and L-strain cells. The culture medium used was prepared from high glucose DMEM supported with 10% fetal bovine serum (FBS, Germany) and 1% penicillin/streptomycin (Lonza, Belgium). The cells were produced under aseptic conditions in an incubator containing 5% CO_2_ and 95% humidity at a temperature of 37°C and cells which reached 80–90% density were planted in wells.

### Quantitative Evaluation of the Cytotoxic Effect

Cell concentration was determined with the Trypan Blue staining method. In this method, after mixing the cell suspension with 0.4% Trypan Blue (Sigma, NY, USA) at a ratio of 1:1, it was placed on a Thoma slide and the cell count was performed under a microscope. Dead cells were observed as a blue colour as the cell membranes had been lost and the viable cells were observed to be clear as they had not taken the stain.

By counting all the parts in the count area, cell concentration (cell/ml) was calculated using the following formula:

A × DF × 104 (A = number of cells counted in the count area, DF = dilution factor).

When cell viability was calculated as 95–99%, a cell suspension was prepared as 105 cell/ml and 100 µl was added to each well of a 96-well microplate (104 cell/well). To hold the cells to the surface, after incubation of the microplates for 24 h, the medium in the wells was removed with a micropipette. Then 100 µl suspension of the prepared test material at four different dilutions (0.0001%, 0.001%, 0.01%, 0.1%) for unpolymerised form and four different timings (after 6 h, 1 days, 3 days and 7 days extraction) for polymerised form were added to the culture medium and the microplate organisation was formed for evaluation of dose-related cytotoxicity. The same procedures were applied in the same way in a total of three microplates and these were incubated for three different time periods (24 hrs, 48 hrs, 72 hrs) at 37°C in a humid medium with 5% CO_2_. Thus, the study scheme was formed for the evaluation of the time-related cytotoxic effects of the test materials on L929 cells.

Following incubation, morphological examination of the cells was applied under an optic microscope lit from below (Olympus IX71, Tokyo, Japan) at ×10 magnification and photographs were taken with a digital camera (Olympus C-400) attached to the microscope. Then the MTT test was applied to determine the rate of live cells. The group to which no test material had been added was used as the control group. After removal of the culture medium containing the test materials, 100 µl serum-free nourishment medium and 10 µl MTT solvent were added to the wells. After incubation for 3 h, the medium over the samples was removed and 200 µl isopropanol (including 0.04 mol hydrochloric acid) was added, then left until the crystal formation dissolved. At the end of this period, the cells were measured at 570 nm with a microplate reader (BioTek-µQuant) spectrophotometer, taking optic density of 690 nm as reference.

The optic density (OD) values in the wells processed with the adhesive agents were compared with the OD values of the control wells and the vitality values (%) for each dilution of the materials were calculated using the following formula:

Vitality (%) = OD (sample) / OD (control) × 100.

### Statistical Analysis

Statistical evaluation of the vitality data (%) obtained in the MTT test was made using Graphpad Prism 5 software. To evaluate the effect on cell vitality of the different doses and incubation periods applied to the test materials, two-way variance analysis was applied to repeated measurements. In groups where statistical significance was found, to determine from which agent and interaction the difference originated, the Bonferroni post-test was applied (p = 0.05).

## Results

### Results of the MTT Test of the Form of the Materials Before Polymerisation

**Fig 1:**
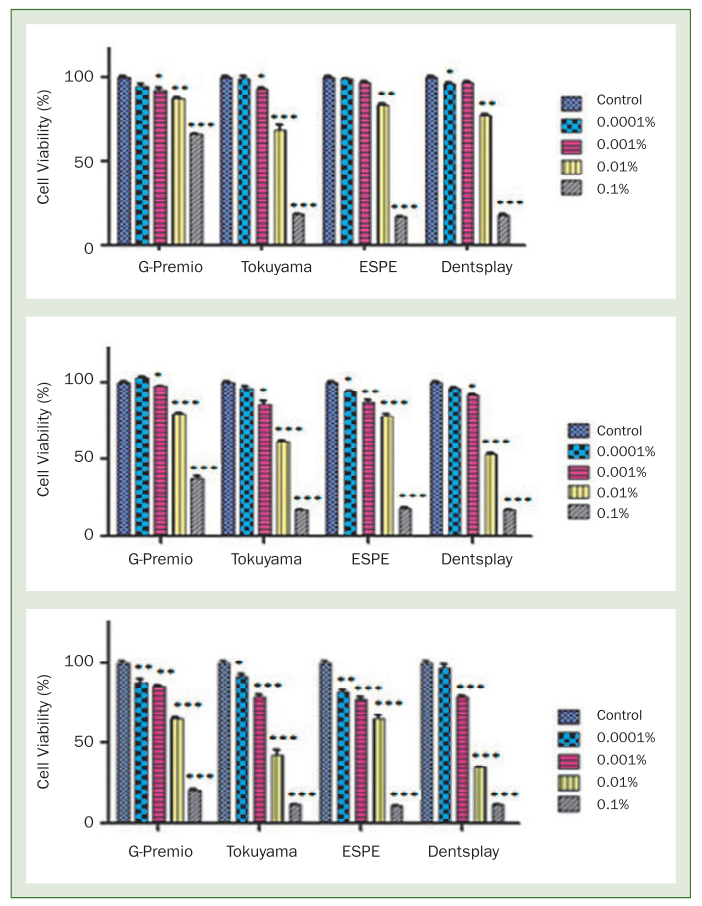
A graphic showing the dose-dependent effect of the test materials on L929 cells after 24, 48 and 72 h incubation period (* p < 0.05, * p < 0.01, *** p < 0.0001).

MTT test, one of the methods of determining cell viability, was applied to evaluate the cytotoxic effects of the test materials in vitro. For this purpose, L929 cells were incubated for 24, 48 and 72 h with four different doses of test materials (0.0001%, 0.001%, 0.01% and 0.1%). Graphs depicting the % viability values of non-polymerised test materials dependent on the duration of the dose and incubation are given in Figure 1. The averages represent the results of two independent experiments in which each variable is made in three replicates.

When the MTT data of GPB adhesive material were statistically evaluated with two-way variance analysis, it was clearly seen that with an increase in the dose and incubation period, the vitality percentage significantly decreased in the L929 cells treated with GPB. An increase in incubation period at low doses was seen to affect cytotoxicity less than at high doses. Even at the lowest dose of 0.001% tested at 24, 48 and 72 h, a statistically significant decrease in vitality percentage was determined compared to the control samples (p < 0.0001).

When the MTT data of TUB adhesive material were statistically evaluated with two-way variance analysis, with the exception of the lowest dose applied at 24 h and 48 h incubation, there was seen to be a statistically significant difference between all the other doses and the control samples (p < 0.0001). All the doses applied at 72 h incubation were seen to be significantly different from each other and from the control samples. Thus it was determined that starting from 0.0001% concentration, the percentage vitality significantly decreased in incubation periods of 24, 48 and 72 h (p < 0.05).

When the MTT data of 3M-E adhesive material were statistically evaluated with two-way variance analysis, there was seen to be a statistically significant difference in the high doses of 0.1% and 0.01% concentrations incubated for 24 h from the control samples, while there was no statistically significant difference in the low doses. Thus it was determined that starting from 0.01% concentration at 24 h incubation, the percentage vitality significantly decreased (p < 0.05). In the 48 h and 72 h incubation periods, all the doses applied were seen to create a significant difference from the control samples, and with the exception of the two lowest doses, from each other (p < 0.05). Accordingly, from the lowest dose tested at 48 h incubation, and from the 0.001% concentration onwards at 72 h incubation, the percentage vitality was determined to significantly decrease (p < 0.05).

When the MTT data of PBU adhesive material were statistically evaluated with two-way variance analysis, the greatest decrease in vitality was seen from the second highest dose onwards. In the 24 h incubation period, the percentage vitality of the two highest concentrations of 0.1% and 0.01% were determined to decrease significantly (p < 0.05). At 48 h and 72 h incubation, percentage vitality significantly decreased from 0.001% concentration onwards (p < 0.05).

### Results of the MTT Test of the Form of the Materials After Polymerisation

**Fig 2:**
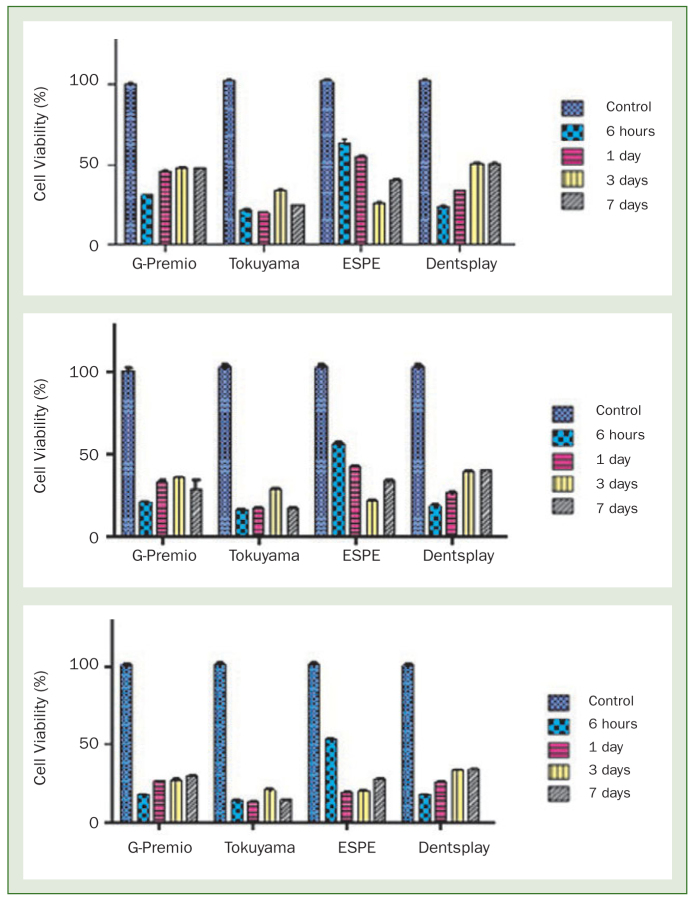
A graphic showing the extraction time-dependent effect of the test materials on L929 cells after 24, 48 and 72 h incubation periods.

To evaluate the cytotoxic effects of the polymerised materials, samples were obtained from L929 cells at the end of four different extraction periods (6 h, 1 day, 3 days, 7 days) and these were incubated for 24, 48 and 72 h. The graphs showing the effects related to the extraction period and incubation period of the test materials are shown in Figure 2. The mean results represent two independent tests performed three times for each variable.

When the MTT data of 3M-E adhesive material were statistically evaluated, it was seen that with an increase in the incubation period, the vitality percentage significantly decreased in the L929 cells treated with G-Premio, and this effect was in inverse proportion to the extraction period. In the samples tested at the end of all the extraction periods, the percentage vitality at the 24, 48 and 72 h incubation periods was determined to significantly decrease compared to the control samples (p < 0.0001). When all the incubation periods were compared, the greatest decrease in cell vitality was determined in the 6 h extraction period. There was a statistically significant decrease in the cytotoxic effect after 6 h but when the 1-, 3-, and 7-day extraction periods were compared, no statistically significant difference was determined.

When the MTT data of the TUB adhesive material were statistically evaluated, there was seen to be a statistically significant decrease in the vitality percentage at all the extraction periods tested, and the greatest decreases were determined in the 6 h and 1 day extraction periods. In the samples tested at the end of all the extraction periods, the percentage vitality at 24, 48 and 72 h incubation periods was determined to significantly decrease compared to the control samples (p < 0.0001). Although the highest toxic effect was seen in the 6 h and 1-day extraction periods, no statistically significant difference was determined between them (p > 0.05). Despite a reduction in the cytotoxic effect in the 3-day extraction period, with the exception of the 24 h incubation period, the cytotoxic effects of the 6 h, 1-day and 7-day extractions were determined to be similar.

When the MTT data of the 3M-E adhesive material were statistically evaluated, there was seen to be an increase in cytotoxicity in direct proportion to the extraction period up to the 7-day extraction and the greatest reduction in vitality was seen in the 3-day extractions. A statistically significant difference was seen in the 24, 48 and 72 h incubation periods of all the tested extractions from each other and from the control samples (p < 0.001). The group with the greatest decrease in cell vitality was the 3-day extraction at 24 h and 48 h incubation periods, while the 1- and 3-day extractions at 72 h incubation were determined with similar rates of cell vitality.

When the MTT data of PBU adhesive material were statistically evaluated, the vitality percentage significantly decreased in the L929 cells treated with Dentsply with an increase in the incubation period, and this effect was in inverse proportion to the extraction period. At the end of all the extraction periods, the percentage vitality of the samples at 24, 48 and 72 h incubation was determined to have significantly decreased compared to the control samples (p < 0.0001). When all the incubation periods were compared, the greatest reduction in cell vitality was determined in the 6 h extractions. The cytotoxic effect decreased significantly after 6 h, but there was no statistically significant difference when compared with the 3- and 7-day extraction periods (p > 0.05).

### Conclusions on the Effect of Test Materials on l929 Cell Morphology

**Fig 3:**
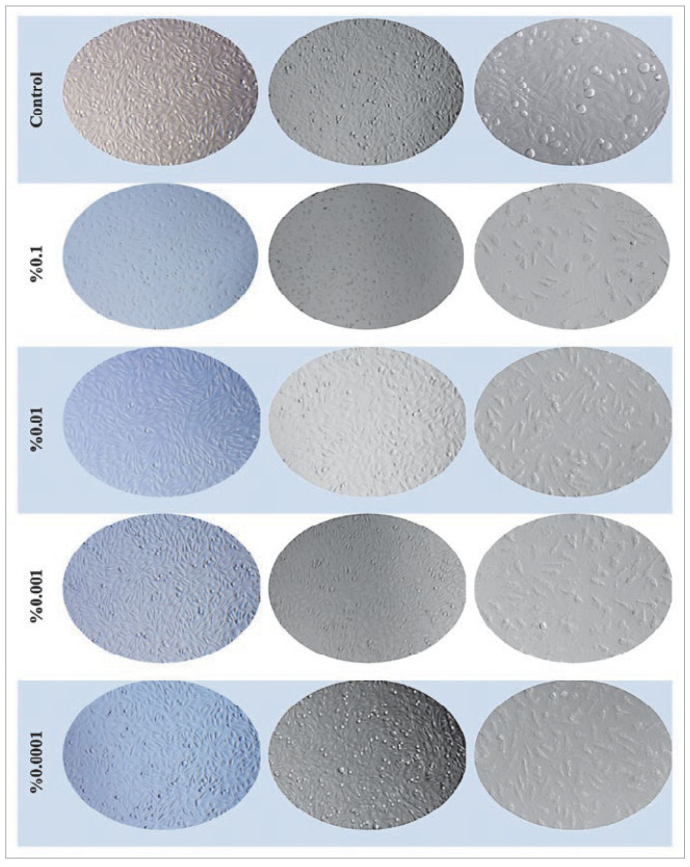
Time and dose-dependent morphological changes on L929 cells treated with unpolymerised PBU.

Microscopic images of the effects of PBU on cell morphology and proliferation, which are generally identified as the most cytotoxic material according to the data obtained before the polymerisation of the test materials, are shown in Figure 3.

When microscope images of L929 cells treated with the test materials in the above conditions are examined in general, it was found that each test material reduced cell proliferation in a dose and time-dependent manner and that the same maximum dose (0.1%) of the test material of each of the test materials resulted in a statistically significant time-dependent decrease in cell adhesion to the surface and that the cell membranes in the highest first two doses of each of the test materials. The deterioration in their structure is clearly visible.

**Fig 4:**
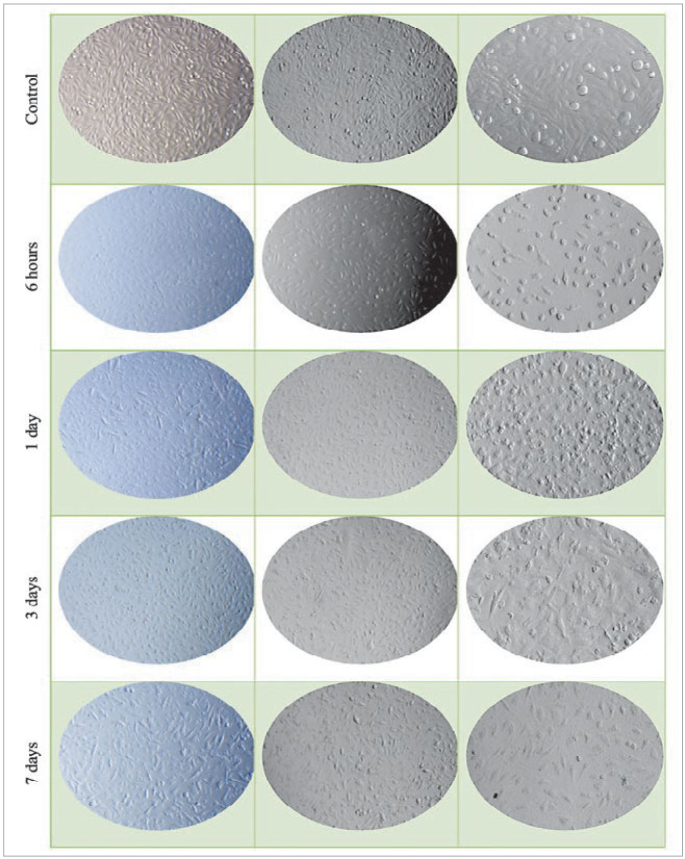
Incubation time and extraction time-dependent morphological changes on L929 cells treated with polymerised TUB.

The microscopic images of TUB’s cell morphology and its effects on proliferation, generally identified as the most cytotoxic material according to the data obtained after polymerisation of the test materials, are shown in Figure 4.

## Discussion

With increasing societal awareness, dentistry patients now demand longer lasting, more aesthetic and more functional restorations. Minimally invasive dentistry with the aim of being able to provide the best aesthetics and function with the least tissue loss and to be able to develop materials which best mimic dental tissue, have formed the basis of rapid developments in adhesive restorative systems. As a result of studies to be able to develop material properties, many products have been presented, sometimes without sufficient laboratory and clinical testing. The ideal restorative material should not only have appropriate mechanical and physical properties, but the necessity for it to be biologically compatible with the tissue with which it is in contact must not be ignored. Therefore, before clinical use of adhesive restorative materials, they must pass through a series of test protocols to determine biocompatibility.

Biocompatibility experiments are used to determine the cytotoxicity of substances to be used as medical devices or materials (ie, if they possess potential toxic effects which may occur before they are used on humans). Cytotoxicity tests are based on the provision of an environment of biological response of mammalian cells inside or outside the body, using appropriate biological parameters. The correct selection of one of these recommended tests or several in parallel is of great importance in biocompatibility studies. In vitro cytotoxicity tests, which are often used for biomedical materials, include qualitative and quantitative evaluation of damage which may occur in cells which are in contact with test and control substances or extracts. In the context of ISO10993-5, there are three qualitative analyses (L929 Elution test, Direct contact test, and indirect contact test) and three quantitative analyses (Neutral Red Uptake test, V79 colony formation test and MTT and related tests).^27^

The MTT test is an effective method used in the determination of cell vitality. This method is based on the principle that the tetrazolium ring can be fragmented by MTT staining of the mitochondria of healthy cells. This method was selected for use in this study as it is a frequently used cytotoxicity test that is simple, rapid and objective.^13,19^ The MTT evaluation procedures require the exposure of the toxin to the test cell line to be 24 h following at least 4 h of incubation. In addition, although this test is a very sensitive test, it requires a waiting time between 52 h and 72 h to complete. Incubation times were determined by taking the MTT test time into consideration.^16^

In a study by Lee et al in 2016, the cytotoxic effects of six different one-step self-etch adhesives were investigated and according to the MTT analysis, Adper Easy Bond and Clearﬁl SE primer (CS) showed the lowest cell vitality.^13^

Ozen et al (2005)^17^ examined the cytotoxic effects on gingival fibroblasts of four different bonding agents and showed that no toxic effect was formed in the first 24 h. At the end of 72 h, the highest cell count was determined in Prompt-L-pop, and the lowest in Pekabond.

In the current study, all the adhesives at low concentrations at the end of a 24 h incubation period showed a similar, low effect. At high concentrations, TUB, 3M-E and PBU showed similar effects while the effect of GPB was seen to be much lower than the others. At the end of a 48 h incubation period, while a dose-related effect in all the adhesives was seen to have significantly increased, the most statistically significant reduction in vitality was seen at high concentrations. In this context, as at 24 h, the material with the lowest cytotoxicity was GPB, and 3M-E and PBU showed similar rates as the most cytotoxic materials. At the end of the 72 h incubation period, the effect was seen to significantly increase related to dose in all the adhesives. At low concentrations, 3M-E was more cytotoxic, while at high concentrations, the effect of Dentsply material was determined to be higher.

In the evaluation of the polymerised materials, TUB could be said to be generally the most cytotoxic material at the end of the 24 h incubation periods. As a result of 6 h extraction, TUB showed the most cytotoxic effect, GPB and PBU were similar and 3M-E was seen to have the least effect. After 1-day extraction, the material cytotoxicity was TUB>PBU>GPB>3M-E, after 3-day extraction, the order was 3M-E>TUB>G-PB>PBU, and after 7-day extraction, TUB>3M-O>GPB>PBU. The most cytotoxic material at the end of the 48 h incubation period was TUB and PBU was seen to be the adhesive with the least effect.

In a study by Reddy, which determined the cytotoxicity of hydroxyethyl methacrylate and bisphenol alpha monomers of adhesive materials, the lowest cytotoxicity value of 0.3125 mg/ml was determined in Clearfil SE Bond. The highest toxic values were recorded at the end of 48 h.^19^ In another study that examined elements, bisphenol A glycidyl methacrylate monomer (bis-GMA) was determined to show the highest toxicity, followed by dimethacrylate and triethylene glycol dimethacrylate (TEGDMA). Hydroxyethyl methacrylate (HEMA) showed moderate level toxicity.^13^

Demirci et al (2008) reported that the dentine primers and dentine adhesives of Clearfil SE Bond and Clearfil Protect Bond decreased cell vitality in a dose-related manner.^7^ Total-etch bonding systems have been proven to be more toxic than self-etch systems in previous studies.^1,4^ Among the four types of self-etch adhesive used in the current study, the toxicity of the bonding agent hardened without light was found to be higher.

In a study by Kierklo et al^9^ of the cytotoxicity of adhesive materials, Adper Single Bond^2^ and Heliobond were reported to have a cytotoxic effect on hepatocyte growth factor cells. Sun et al^22^ investigated the cytotoxicity of one-step self-etch dental adhesives and the highest cytotoxic effect was observed in G-Bond and the lowest in I-bond. Five different bonding agents were examined in respect of cytotoxicity by Cal et al^3^ and the efficacy of Clearfil S3 Bond was found to be significantly different from that of the other materials.

In another study by Kusdemir et al^12^ in which the cytotoxicity of six self-etch bonding systems were evaluated with direct and indirect tests, Clearfil SE Bond (CSE), and Clearfil Protect Bond were seen to be less toxic than the other adhesives. Using the dentine barrier test of one-step self-etch adhesives to evaluate cell vitality, Kim et al^10^ reported the highest cell vitality in Futurabond D9C and the lowest in Bond Force. In another study by Sengun et al that evaluated the cytotoxicity of adhesive materials, Adper Prompt Self-Etch and G-Bond (GB) were observed to be more toxic than the other materials tested.

## Conclusion

In this study, the potential cytotoxic values of four different universal bonds were compared, before and after polymerisation. Although the most cytotoxic material before polymerisation was PBU, after polymerisation PBU was determined as the least toxic material. TUB, which is polymerised with mild air without light, was the adhesive material determined with the highest cytotoxicity. Therefore, PBU should be preferred over TUB to be able to provide clinically long-lasting restorations.
